# Are Exam Questions Known in Advance? Using Local Dependence to Detect Cheating

**DOI:** 10.1371/journal.pone.0167545

**Published:** 2016-12-01

**Authors:** Stefan Zimmermann, Dietrich Klusmann, Wolfgang Hampe

**Affiliations:** Department of Biochemistry and Molecular Cell Biology, University Medical Center Hamburg-Eppendorf, Hamburg, Germany; George Washington University, UNITED STATES

## Abstract

Cheating is a common phenomenon in high stakes admission, licensing and university exams and threatens their validity. To detect if some exam questions had been affected by cheating, we simulated how data would look like if some test takers possessed item preknowledge: Responses to a small number of items were set to correct for 1–10% of test takers. Item difficulty, item discrimination, item fit, and local dependence were computed using an IRT 2PL model. Then changes in these item properties from the non-compromised to the compromised dataset were scrutinized for their sensitivity to item preknowledge. A decline in the discrimination parameter compared with previous test versions and an increase in local item dependence turned out to be the most sensitive indicators of item preknowledge. A multiplicative combination of shifts in item discrimination, item difficulty, and local item dependence detected item preknowledge with a sensitivity of 1.0 and a specificity of .95 if 11 of 80 items were preknown to 10% of the test takers. Cheating groups smaller than 5% of the test takers were not detected reliably. In the discussion, we outline an effective search for items affected by cheating, which would enable faculty staff without IRT knowledge to detect compromised items and exclude them from scoring.

## Introduction

In 1986 a questionnaire survey on cheating 23.7% of college students reported to have cheated at least once on major exams [1]. Cheaters tended to have lower grade point averages with a correlation between cheating and GPA of r = -.23. Low grade students seem to be least affected by threats and most inclined to justify cheating by neutralization (denial of responsibility, denial of the victim, denial of injury, condemnation of the condemners, and appeal to higher loyalties). In an overview of more recent research on cheating McCabe et al. reported that cheating (as admitted in a questionnaire) went up from 39% in 1963 to 64% in 1993 [2]. They found institutions with an honor code of academic integrity to be less troubled by cheating students. Therefore an institution should communicate that it really cares about fairness.

Cheating needs to be detected mainly for two reasons particularly for tests associated with program admission: (1) Applicants who invested much effort to prepare for the test would feel betrayed if they were rejected because cheaters surpassed their scores and pushed them back in the rank order below the point of rejection. (2) Cheating undermines the predictive validity of admission or licensing tests.

There are different possibilities how to cheat [3, 4]. During the last years new technical equipment has been developed and sold with cheating in mind: cameras that are connected to a cell phone to transmit questions to an external expert at the beginning of the testing time who returns the answers to the earpieces of a group of participants before the end of the test. Even a simple cell phone is enough to capture pictures of exam material in order to cheat [5].

Another way is to get access to some or all items before the test session. In the United States in 1997, the National Board of Medical Examiners (NBME) withheld the scores of more than 20,000 medical students because “some examinees could have had access to the exam before the administration of the test”[6]. In 2004, the Medical Council of Canada was warned that test questions for the Canadian medical license examination had been sold [7].

In simultaneous testing all test items are administered to all test takers at the same time. This setting is characteristic for university and licensing exams and many admission tests. Tests are typically composed of a set of newly developed items and a set of old items repeated from earlier versions. Items are reused mainly for three reasons: (1) reusing items with sound psychometric properties improves the quality, (2) creating new items is costly, and (3) test equating requires item overlap between tests. The reused items are especially vulnerable to item preknowledge (IP), since they might be memorized and passed on to future test takers. For instance, a test preparation company sent instructed agents to memorize questions of the 1994 Graduate Record Examination [3].

In this study we present a procedure by which items affected by cheating can be identified in the dataset of student responses.

### Detection of item preknowledge

In Computerized Adapted Testing (CAT), repeated utilization of items is unavoidable, especially when the item pool is small. Current research is mostly concerned with the development of sophisticated statistical methods to detect test takers who had used IP in CAT. Most such methods exploit the difference between new items which have not yet been exposed and repeated items which *might* have been memorized by some test takers and then passed on to future test takers who might encounter some of them in their own test. McLeod describes a Bayesian method to detect IP [8]. With each new item response, a prior probability that the test taker has used preknowledge is updated by assessing the person´s model fit to an underlying statistical model—an Item Response Theory (IRT) model in which the guessing parameter of a 3PL model is enhanced to include IP. The final measure is a log odds ratio reflecting the suspicion that the test taker is cheating. With this indicator, a sensitivity of 80% is achieved at a specificity level of 97% in a test with 28 items of which 15 are compromised [8]. Another model is the deterministic gated IRT model (DGM) [9], which decomposes test taker´s performance into two distributions, one for the “true ability” and one for “cheating ability”. Cheaters are identified by their score gain due to cheating.

For the detection of IP in large data sets, Belov suggests a multistep approach [10]. First, all available information about the test takers is used to investigate all possible meaningful subsets to identify groups that possibly could have colluded in gaining item preknowledge (applicants who went to the same high school, who come from the same geographic region, or who went to the same test preparation, etc.). Corresponding groups are identified by comparing the person-fit distribution to the overall sample. Second, for each affected group a plausible subset of compromised items is revealed by an optimization algorithm.

If the proportion of potentially compromised items is large relative to the item set guaranteed to be uncompromised, even highly sophisticated statistical methods are ineffective, especially if the number of affected items and the number of cheating respondents is small [8]. If only few cheaters are present these are virtually undetectable. The prospect to detect cheaters increases as both the proportion of cheaters and the proportion of preknown items increases up to a point where cheating is so prevalent that detection becomes harder again, because virtually everyone is cheating. In these cases, however, the test score distribution is noticeable aberrant.

In summary, detecting IP seems to require a high level of IP in the dataset and a high level of statistical sophistication.

Almost all these efforts to detect IP focus on persons. Here, we present an approach that works instead to detect the compromised items. It is simple enough to be used as part of a regular Item analysis with an IRT model [11] and will detect IP at a fairly low level. The subject of our analysis is the Hamburg Natural Science Knowledge test (HAM-Nat) [12]. This test has been used yearly since 2008 for the simultaneous selection of medical undergraduate students in Hamburg, Berlin, and Magdeburg, Germany. Items are drawn from biology, chemistry, physics and mathematics covering the scope of high school teaching. Every year, a new test version is composed of 40 new items and 40 old items from previous test versions. In recent years, the number of internet postings about the items of the HAM-Nat have increased, and preparation courses offered by commercial firms have gotten more sophisticated. So an incentive for item theft might exist.

### How would item preknowledge affect item parameters, item fit and local dependence?

If an item is preknown, changes are to be expected in the following indicators which are generated by an IRT model (for details see de Ayala [11] and Embretson and Reise [13]):

- *Item difficulty* should decrease because preknowledge produces an excess of correct responses.
- *Item discrimination* should decrease because in the subgroup of preknowing test takers almost all items preknown in advance are correct and therefore not related to ability as estimated by the uncompromised part of the item set.
- *Item fit to the IRT model* should decrease because compromised items will be frequently correct when the 2PL model predicts an incorrect response.
- *Person fit to the IRT model* should decrease because cheating test takers will tend to produce an anti-Guttman-pattern (see for Guttman-scaling [14]): More often than other test takers easy items are failed and hard items are answered correctly—a pattern with low likelihood.
- *Local dependence (LD) of item pairs* should increase. In an IRT model the correlation between two items must depend exclusively on the relation both items have with the ability they measure. If the level of ability is held constant (at a fixed location on the ability scale), the correlation should disappear. In other words: After the ability measure is excluded the residual correlation between two items should become zero. This requirement is called *local independence*. If it is violated and items are locally dependent, test information and reliability will be overestimated [15]. Local dependence (LD) increases whenever items have some property in common which is independent of the ability dimension. This might be similarity of content and, most important for us, the property of being preknown to some test takers (IP).

Each of these indicators might change for reasons other than IP. For example item difficulty may drop not only because of IP, but also because the new cohort was better prepared than the preceding cohort, or the new cohort took part in a natural science curriculum particularly well tuned to the content of the item [16]. Item discrimination may decrease because each new test contains a large proportion of new items which has shifted the centroid of the item set, thereby slightly changing the meaning of the scale, e.g. nudging it more toward biology, or physics, or chemistry. Item fit to the IRT model may be reduced because of guessing or construct-irrelevant features, e.g. ambiguous wording. Person fit to the IRT model may be reduced because of language difficulties, sloppiness, test fatigue, island knowledge, running out of time, etc. [17]. High LD may be caused by similarity in content, item chaining, test speediness, hidden dimensionality and many other sources of disturbance.

Will item preknowledge stand out against competing sources of change? Fortunately a unique profile is expected: IP not only affects one coefficient or two, but all coefficients simultaneously. If a repeated item shows a simultaneous drop in (1) discrimination, (2) difficulty and (3) item fit together with (4) an increase in local dependence, then IP is probably the cause and alternative causes, albeit still possible, are hard to conceive.

## Method

We designed a simulation study for the following imagined scenario: What if a copy of the 2012 HAM-Nat had been stolen and used clandestinely in preparation courses attended by, say 10% of applicants who took the next test in 2013? The eleven items which are to be repeated in the 2013 version would be preknown by 92 of 920 test takers. Based on a real data set from the 2013 HAM-Nat, we generated an altered data set with simulated test takers with item preknowledge:

A randomly selected group of 10% of test takers was assigned to possess item preknowledge for the sake of the simulation (simulated cheaters). We slightly oversampled low performers in the HAM-Nat because we expected motivation to seek preknowledge to be inversely related to ability. Technically a random variable (mean = 0, SD = 1) weighted by 3 was added to the z-standardized test score; test takers with the lowest 10% on this score were selected. This gave rise to a correlation of r = -.19 between HAM-Nat test scores and membership in the cheater group (yes/no). This is still a conservative oversampling—in the study of Haines et al. a correlation of -.23 was found between GPA and cheating behavior [1].

In the subgroup of simulated cheaters 90% of the responses to the 11 repeated items were set to “correct”. We did not change 100% of responses in order to account for mistakes and incomplete memory recall. All other items were left unchanged. This is our definition of an item set compromised by preknowledge.

The ethic commission board (Ethik-Kommission der Ärztekammer Hamburg, PV4983) has approved that our admission research in general does not constitute research with human subjects (“kein Forschungsvorhaben am Menschen”) in a clinical sense. This holds true particularly for the present study in which the observational units are items and not persons. The present study is part of a larger project that has been approved by the dean of the Hamburg medical faculty (statement of ethical considerations “development of admission procedures at Hamburg Medical School”). Basic socio-demographic information was collected but was not part of the present analysis. In accordance with the ethical principles of psychologists and code of conduct [18], informed consent may be implied “because testing is conducted as a routine educational, institutional or organizational activity (e.g., when participants voluntarily agree to assessment when applying for a job)” (p. 12) or dispensed “where research would not reasonably be assumed to create distress […] and involves the study of normal educational practices […] conducted in educational settings” (p. 10). Still, we obtained written informed consent from our participants who ranged in age from 16 to 35. For the very few underage participants we did not reach out additionally for their parents’ or guardians’ consent considering the reasoning above. Only abstract anonymized response patterns that do not allow the identification of persons were analyzed in the simulation of item preknowledge.

We will first present the results of the simulation run for the HAM-Nat 2013 with10% simulated cheaters and 11 repeated items in detail (abbreviated as HN2013;10%;11) and then shortly describe seven further simulation runs to get some insight into the detection rate and its generalizability. The IRT parameters were estimated with the software IRTPRO 2.1. Data analysis focuses on changes (Δ) in item difficulty, item discrimination, item fit and local dependence between non-compromised and compromised data sets.

### Necessity of test equation

In order to attribute such differences in item difficulty, item discrimination, item fit and person fit to IP, both tests have to form a common scale. Therefore test equivalence has to be assured beforehand by equating. To do this, the anchor set of items used for equating should be free of IP. But IP cannot be excluded *before* the tests are equated (as any source of differential item functioning [19]).

At first glance, this seems to implicate an unsolvable quandary. However there are two ways out: (1) assuming a priori that the concurrent cohort´s ability is about the same as the preceding cohort´s ability, (2) relying on an indicator such as LD that needs no baseline from preceding test versions.

The first way out requires an argument by continuity: If the ability of applicants did not change from year to year in the past, it seems reasonable to assume that it will be roughly the same in a new test, too. Of course IP would make the test easier, but this effect would be small. In our simulation for 2013 (HN2013;10%;11), mean item difficulty only dropped from 0.13 to 0.07. The ability change is negligible, thus equating is not necessary, and differences between coefficients can be used without modification.

The second way out relies on local dependence, since LD is the only indicator that can be interpreted without prior test equating. If the preceding test did fully conform to the ideal IRT requirement of local independence, the baseline of LD would be zero for every item, and any LD in the following test would stand out as aberrant. However, in reality most tests are burdened with an amount of tolerated deviation of the model. In the case of the HAM-Nat, which is modelled as one single dimension, it is some hidden dimensionality. The set of old items might well be purged of items with intolerable LD, but with the set of new items this is not possible. So the baseline is not zero LD and therefore a difference reflecting LD change from one test occasion to the other needs to be computed. Still, this requires no prior equating and makes LD an attractive indicator for the detection of item preknowledge.

LD was measured for each *item pair* as a χ^2^ value reflecting the discrepancy between expected and observed values in the cross table of an item pair [20–22]. Chen and Thissen distinguished between “surface LD” and “underlying LD” [20]. Surface LD arises whenever the response to one item is simply a copy of the response to the other items. In contrast, underlying LD is mainly LD due to substantial commonality, e.g. hidden dimensionality. LD due to item preknowledge is clearly surface LD, because the response “item correct” is mechanically the same for the whole item set preknown. In the case of surface LD, the coefficient LD χ^2^ performs as well as more sophisticated coefficients like Q_3_ [20].

### Determination of the change in local dependence

We computed the total local dependence of an item LD χ^2^_count_ as the count of all LD-values of this item exceeding the threshold of LD χ^2^ = 3.86. This threshold corresponds to an alpha level of .05. In an 80 items test, LD χ^2^_count_ of an item ranges between 0 and 79.

## Results

**Fig 1:**
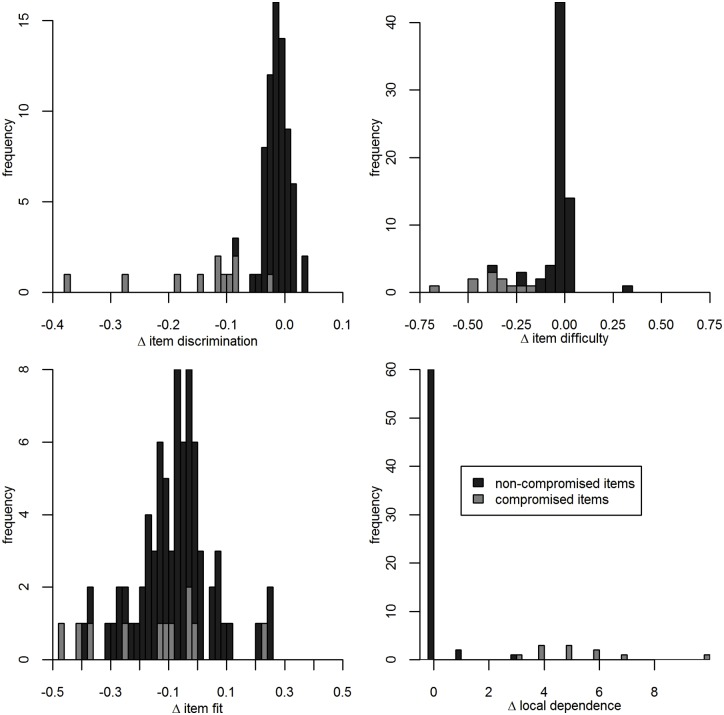
Shift in item parameters, item fit and local dependence after items have been compromised by preknowledge (HN2013;10%;11 versus HN2013;0%;11).

In the compromised item set, item discrimination and item difficulty decrease markedly while local dependence increases (Fig 1). There is no clear pattern in the change of item fit.

**Fig 2:**
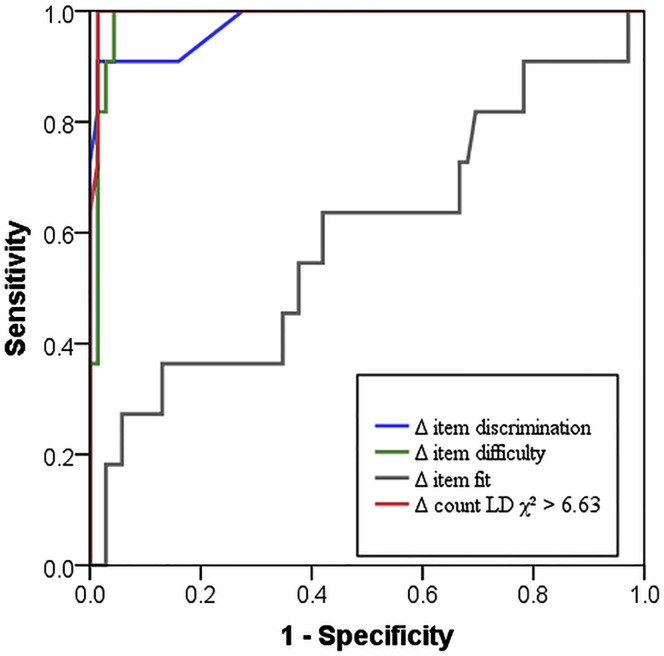
Receiver Operating Characteristic curve for selected indicators for item preknowledge in the 2013/10%;11 simulation.

In reality, compromised items are not known beforehand and must be determined by the indicators. Therefore cut-off scores with high sensitivity and specificity need to be defined. The receiver operating characteristic (ROC) [23] curves depict this trade-off as the ratio between the proportion of items correctly detected as compromised (sensitivity) and the proportion of false positives (1—specificity) at any cut-off point used for detection (Fig 2). Every increase in sensitivity has to be paid for by an increase in false positives. This is reflected by the area under the curve: The minimum area is 0.5 for a random guessing strategy, the maximum is 1.0 corresponding to detection with absolute certainty and without false positives. In the current simulation, the LD χ^2^-count identifies all compromised items without error. Therefore, the corresponding ROC curve is located at the outer edge of Fig 2. Shifts in item discrimination and item difficulty allow a good but not perfect identification of the preknown items, as the area under the curve amounts to .87 and .95, respectively. In comparison, a shift in item fit performs just slightly better than chance (.58). Therefore, we excluded item fit from further analyses.

### A synthetic indicator: Suspicion for IP (SIP)

The three indicators Δ difficulty, Δ discrimination, and Δ LD are expected to change *simultaneously*. Therefore a combination should provide even more discriminative power. This combination should be multiplicative to be sensitive to simultaneity and not to allow for a compensation between the indicators. Technically we transformed the distribution across items of each coefficient to a mean of 10 and a standard deviation (SD) of 1 and multiplied the resulting scores to a three-term product. This we call the “Suspicion for IP” indicator (SIP). The SIP indicator should be superior to any of its constituents. In the following simulations we calculated SIP for each item (for mean values in the compromised and non-compromised items, SDs, and effect sizes see S1 Table).

### Additional simulations

Assuming that 10% of test takers come prepared with item preknowledge is perhaps not realistic—in real life the proportion may be lower. Therefore, we repeated the simulation with the 2013 HAM-Nat, setting the proportion of cheaters to 5%, 2.5% and 1%. Additionally, we performed equivalent simulations with the 2014 HAM-Nat with 1030 test takers, assuming that the 2013 HAM-Nat was leaked. The test versions for 2014 and 2013 have seven items in common, so the percentage of compromised items was only 9%.

Table 1 shows the sensitivity of coefficients in eight simulations runs with various proportions of test takers with IP (for mean values, SDs, and effect sizes see S1 Table). The rate of false positives (1-specifity) was fixed at 5%. Results: (a) Sensitivity is perfect or nearly perfect for all indicators if 10% of the test takers possess IP. (b) A shift in item discrimination is still sensitive with low frequencies of IP, whereas the sensitivity of the shifts in item difficulty and number of LD relations of an item (LD χ^2^ count) drops severely when the proportion of IP test takers drops below 5%. (c) The multiplicative indicator SIP is superior to any of its constituents with only two exceptions in 2014.

**Table 1:** Sensitivity of indicators of item preknowledge if rate of false positives is 5%.

Year	Fraction of cheaters	Δ difficulty	Δ discrimination	Δ LD χ^2^ count	SIP
**2013**	10.0%	1.00	0.91	1.00	1.00
	5.0%	0.36	0.64	0.73	0.91
	2.5%	0.36	0.64	0.09	0.73
	1.0%	0.09	0.64	0.00	0.82*
**2014**	10.0%	1.00	1.00	0.71	1.00
	5.0%	0.86	1.00	0.43	1.00
	2.5%	0.71	1.00	0.29	0.71
	1.0%	0.14	1.00	0.29	0.71

Note: The sensitivity of a test is the proportion of subjects identified correctly as exhibiting the proportion in question (in this case IP).

* SIP is not zero even when ΔLD χ^2^ count is zero, because all three variables entering SIP are transformed to a distribution with a mean 10 and SD 1.

The poor performance of item difficulty and the LD χ^2^- count at low frequencies of test takers with item preknowledge seems to dilute the specificity of the multiplicative indicator SIP. However, SIP has the advantage, of responding more specifically to item preknowledge than item discrimination alone.

### The structure of the LD matrix

**Fig 3:**
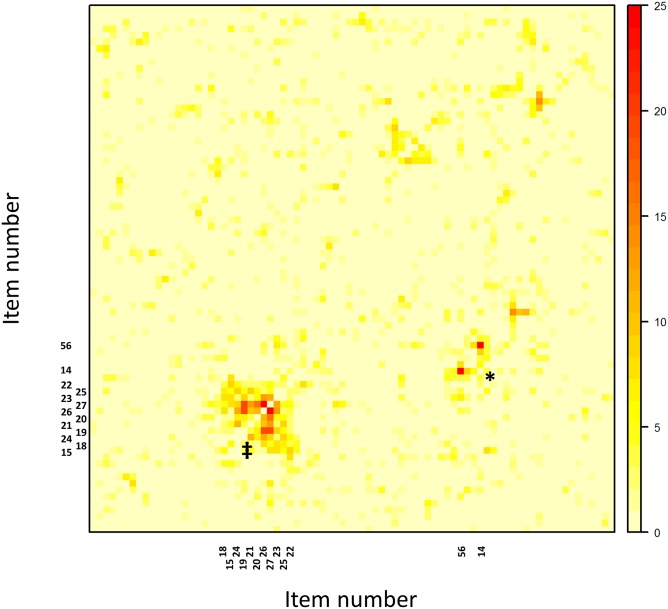
Heat map of LD values in the 80x80 matrix of the HN2013;10%;11 simulation. Note: Each item pair occurs twice. Due to the bond energy algorithm, items on the x-axis are not in the same order as on the y-axis. Therefore the plot is not symmetrically. ‡ 11 items compromised by item preknowledge. * 2 items locally dependent due to item content (item 14 and item 56). Both items are about DNA replication and require the same answer.

The discriminating power of local dependence can be improved. Up to this point, the LD χ^2^- count uses a measure of magnitude for an item´s LD which simply counts how often LD with any other item exceeds a certain threshold. However, not every high LD item pair indicates item preknowledge, which requires multiple LD- relations within a subgroup of (compromised) items. How can we benefit from this restriction when the set of compromised items is not known beforehand? We tried a hierarchical cluster analysis. The compromised items clustered earlier than other items, but there was no gain in accuracy of detection above what could be gathered from the simple LD χ^2^_count_ alone. Next we tried a multitude of methods designed to visualize the similarity of rows and columns in an asymmetrical matrix. A heat map represents the local dependence of each item pair by a color [24] (Fig 3). Best results were achieved using the bond energy algorithm (BEA) [25] which rearranges rows and columns so that each entry is as closely related as possible to its nearest four neighbors.

In this map the compromised item set stands out as a cluster before a background of non-compromised items that looks like a starry sky with randomly distributed minor clusters of small item sets which are locally dependent for other reasons than item preknowledge. The bond energy algorithm brings the 11 compromised items in an unbroken sequence. A core of 5 items is tightly related and surrounded by 6 items with lesser ties. If we had not known the compromised item set beforehand, the heat map would have shown it.

### Analysis of the 2015 HAM-Nat

To apply the proposed analysis to an independent test, we used the 2015 HAM-Nat. In this case we did not know if some test takers had used IP and which items might be compromised. First, we looked at the local dependence between the 80 items in a heat map, and found no peculiar pattern. Second, we calculated for each reused item the SIP value. Three items displayed SIP above 1.5 SD. These items had become easier and overall more locally dependent but not with each other. Furthermore, they originated from test versions of different years. In summary, they did not fit the characteristic pattern for item preknowledge as seen in the simulation studies. Therefore, we have no indication of an item leak for the 2015 HAM-Nat.

## Discussion

Item preknowledge seems to be fairly detectable in simulations of student response data even at low frequencies by item parameters and patterns in the local dependence matrix. Local dependence is particularly valuable because the interpretation of this indicator needs no prior test equating. In our search for papers on the detection of item preknowledge, we found no references to local dependence as a promising coefficient, and, conversely, in the literature about local dependence we only found one marginal note saying that “external assistance” might also lead to local dependence [15].

An indicator combining changes in item difficulty, item discrimination, and local dependence is probably even more sensitive to item preknowledge. However, this requires different tests be equated. This procedure might itself be compromised by item preknowledge. Such a quandary is described in a paper on equating the MRCP(UK) exam (Membership examination of the Royal Colleges of Physicians of the United Kingdom) with a Rasch model [26]. McManus et al. used only some anchor items for equating. The other anchor items were examined to check for a drop in difficulty that might indicate item leakage. None was found. However, if the items used for equating had already been compromised, this procedure would not have given valid results.

This difficulty makes LD the most attractive indicator for item preknowledge, because LD can be used without assuming test equivalence. Of course, there is random LD and there are small pockets of LD for substantial reasons such as content similarity and hidden dimensionality in every test. Still, if the frequency is high enough, item preknowledge seems to produce a suspicious pattern of LD that stands out visibly against the rest of the LD matrix even if only 5–10% of the test takers are cheating.

Monitoring for item preknowledge is part of the routine data analysis of the HAM-Nat. In our item bank, item pairs sharing LD above the .05-level are flagged. This information is used to scrutinize the item list for suspicious sets with elevated LD and to avoid LD in newly composed tests. Suspected preknown items should be excluded from the scoring process and not be used again in future test versions.

Having found a set of items with suspicious LD χ^2^ values, we are in a good position to identify test takers who have used preknowledge. These cheaters will perform much better in the suspicious item set than in the remaining item set, so they can be identified at least with some probability. After this is accomplished, the suspicious item set may again be scrutinized in order to remove items which did not acquire their elevated LD χ^2^ values from item preknowledge and thus would not specifically distinguish cheaters from the rest.

### Standard procedure to check for cheating

An exhaustive checking routine for item preknowledge proceeds like this:

1. Determine if overlapping item sets from previous test versions exhibit excessive LD χ^2^.
2. If not, use these item sets as anchor sets for equating. If yes, then exclude these items from equating or, if this will eliminate too many anchor items, provisionally assume test equivalence and re-evaluate this assumption after the check for IP is completed.
3. Compute the indicator SIP
4. Select items with the highest SIP values.
5. Compute a count of LD χ^2^ above a threshold restricted to this set.
6. Exclude items with a low count—the remaining items are suspicious items.

This routine can be made more specific by subsequent identification of the suspicious test takers and analysis of the item parameters in this group.

Faculty staff is usually not familiar with IRT analyses. Nevertheless are questions reused not only in sophisticated licensing or admission tests, but also in regularly repeated university examinations. To detect item preknowledge in these exams the procedure has to be automatized. Some universities already make use of item management software that is developed in a collaborative assessment network and that allows them to assemble, score and analyze exams. Adding our checking procedure into such software would provide the faculties with a heat map or a list of suspicious items for closer inspection. Compromised items could then be excluded from scoring to neutralize the most likely cheating attempts.

Item preknowledge might be considered as one example for external assistance. Our strategy can also be used to detect other types of cheating behavior that leads to shared item preknowledge e.g. if correct answers are shared via a WhatsApp-group or transferred to a group of test takers via earpieces.

The conclusiveness of our investigation is limited by the small number of simulation runs (eight simulations based on two data sets). Still it shows that it is possible to incorporate a fairly effective check for the detection of item preknowledge into a routine test analysis by just using basic indicators. Such a check might help to safeguard against item preknowledge in admission tests, written exams, tests of cognitive ability and many other fields of application where there is an incentive to cheat.

## Supporting Information

S1 Table Mean differences (SD) and effect sizes d between the real data set and the simulated data set in the non-compromised and the compromised items. (DOCX)
