# Supplementary material for: Are Exam Questions Known in Advance? Using Local Dependence to Detect Cheating
Source: PLoS One. 2016 Dec 1;11(12):e0167545. doi: 10.1371/journal.pone.0167545 (PMC5131967; doi:10.1371/journal.pone.0167545)
Supplement: S1 Table — (DOCX) [file pone.0167545.s001.docx]

| **Cheaters** | Δ **difficulty** | | | Δ **discrimination** | | | Δ **LD**  χ**² count** | | | SIP | | |
| --- | --- | --- | --- | --- | --- | --- | --- | --- | --- | --- | --- | --- |
| **2013** | **non-compr. items** | **compr. items** | **d** | **non-compr. items** | **compr. items** | **d** | **non-compr. items** | **compr. items** | **d** | **non-compr. items** | **compr. items** | **d** |
| 10.0% | .07 (.49) | -.36 (.14) | -.93 | -.01 (.02) | -.14 (.10) | -3.22 | -.07 (.36) | 5.36 (1.91) | 7.13 | 922.6 (99.7) | 1581.0 (311.7) | 4.53 |
| 5.0% | -.02 (.18) | -.16 (.06) | -.83 | -.00 (.01) | -.06 (.06) | -2.56 | -.06 (.34) | 1.27 (1.42) | 2.22 | 939.6 (107.7) | 1441.0 (398.9) | 2.87 |
| 2.5% | .04 (.55) | -.08 (.04) | -0.23 | -.00 (.01) | -.04 (.03) | -2.81 | -.01 (.27) | -.09 (.70) | -0.23 | 973.0 (138.6) | 1172.9 (354.9) | 1.10 |
| 1.0% | .04 (.30) | -.03 (.02) | -0.25 | -.00 (.01) | -.02 (.02) | -1.70 | -.01 (.12) | -.09 (.30) | -0.52 | 976.5 (140.8) | 1137.3 (272.5) | 0.98 |
| **2014** | **non-compr. items** | **compr. items** | **d** | **non-compr. items** | **compr. items** | **d** | **non-compr. items** | **compr. items** | **d** | **non-compr. items** | **compr. items** | **d** |
| 10.0% | .08 (.74) | -.29 (.10) | -0.52 | -.01 (.01) | -.26 (.15) | -6.00 | .03 (.55) | 3.43 (3.05) | 3.47 | 948.3 (106.2) | 1655.6 (498.5) | 4.12 |
| 5.0% | .02 (.18) | -.21 (.18) | -1.28 | -.00 (.01) | -.16 (.10) | -5.57 | .06 (.44) | 1.14 (2.04) | 1.55 | 951.7 (109.9) | 1597.7 (498.8) | 3.71 |
| 2.5% | .03 (.25) | -.07 (.05) | -0.42 | -.00 (.01) | -.10 (.06) | -5.30 | .08 (.40) | .86 (1.68) | 1.31 | 961.8 (127.6) | 1485.9 (558.8) | 2.65 |
| 1.0% | .01 (.11) | -.03 (.03) | -0.38 | -.00 (.01) | -.05  (.04) | -3.41 | .08 (.28) | .86 (1.22) | 1.80 | 960.8 (126.0) | 1500.0 (544.4) | 2.79 |
